# Comparison of negative-pressure incision management system in wound dehiscence: A prospective, randomized, observational study

**DOI:** 10.25122/jml-2019-0033

**Published:** 2019

**Authors:** Mehmet Ali Gök, Mehmet Tolga Kafadar, Serkan Fatih Yeğen

**Affiliations:** 1. Clinic of General Surgery, Health Sciences University, Derince Training and Research Hospital, Kocaeli, Turkey; 2. Clinic of General Surgery, Health Sciences University, Mehmet Akif İnan Training and Research Hospital, Şanlıurfa, Turkey; 3. Clinic of General Surgery, Ali Osman Sönmez Oncology Hospital, Bursa, Turkey

**Keywords:** Surgical site infection, wound dehiscence, negative-pressure wound treatment

## Abstract

Wound dehiscence is a significant problem faced by surgeons after major abdominal surgery. In this study, it was aimed to select the best incision management system to keep the incision edges together and prevent wound opening, and infection by protecting the incision. In this study, 60 patients who underwent abdominal surgery were evaluated regarding their risk of wound dehiscence. In our clinic, high-risk cases of abdominal surgery are performed, the risk factors being ischemia along the incision line, dirty and contaminated wound, obesity, tension on the suture line, traumatization of the wound site, age at onset (> 65), body mass index (BMI) > 30, diabetes mellitus, chronic obstructive pulmonary disease (COPD), immunosuppressive drug users. A prospective study protocol was planned after ASA (American Society of Anesthesiologists) physical status class assignment. Patients were divided into three groups: patients who underwent a postoperative negative-pressure therapy dressing, patients who underwent subcutaneous aspiration drainage, and patients who received standard dressing. The aim of this study was to evaluate the decompensation, surgical site infection, seroma, hospital stay and costs and to evaluate the results in the postoperative period. Sixty patients were randomized (n = 20, for each group). Thirty-one (51%) of the patients were male, and the mean age was 64.3 ± 8.9 (46-85). The mean BMI was 30.45 ± 7.2. There was no statistically significant difference (p≥0.05) between groups in terms of sex, age, and BMI. The ASA score and surgical interventions were similar between the groups. Wound dehiscence rate was 25% (n = 8), 20% (n = 6) and 3% (n = 1) for the Standard Dressing (SD), Aspiration Drainage (AD) and Negative-Pressure (NP) groups, respectively (p <0.017). Duration of hospitalization was 16.45 ± 6.6, 14.3 ± 7.4 and 8.95 ± 2.8 days (p <0.001) for SD, AD and NP groups, respectively. No statistically significant difference was found between the groups regarding other variables (p≥0.05 for all variables). Negative-pressure wound treatment is an easy, fast and practical technique which reduces lateral tension and swelling. It provides perfusion support and helps to protect the surgical field against external sources of infection.

## Introduction

Wound healing is an intricate process where an organ and tissue tries to repair itself following injury. Cytokines, chemokines and growth factors play a significant role in this healing period. The classic model of wound healing is divided into three or four sequential phases: hemostasis, inflammation, proliferation and remodeling. Right after the injury, platelets adhere to the site of damage and become activated. Through activation of the coagulation cascade, the so-called blood clot is formed and bleeding stops. This phase initiates hemostasis. The inflammation phase helps cells and debris to be removed from the injury site. The proliferation phase involves angiogenesis, collagen deposition, granulation tissue formation, epithelialization and wound contraction. Finally, during maturation and remodeling, collagen takes the lead and ends the healing process [1]. Some factors remain a major obstacle for wound healing, primarily venous or arterial disease, diabetes, advanced age, and various metabolic disorders. Factors controlling the efficacy, speed and manner of wound healing can be classified as local and systemic. Mechanical factors, edema, ischemia, necrosis, foreign bodies and low oxygen levels are local factors, whereas inadequate perfusion, inflammation, diabetes, nutrients, metabolic diseases, immunosuppression, connective tissue disorders and smoking are systemic factors. Adequate blood flow is one of the significant factors that contribute to wound healing. Insufficient blood flow can cause chronic wounds, depending on the ischemia degree. This type of damage may be a candidate for infection, necrosis and wound dehiscence. Wound dehiscence is a frequent clinical entity following major abdominal surgeries resulting in considerable problems [2]. In the present study, we tried to evaluate the best incision management system for bringing wound edges together, preventing infection by covering the injury to eliminate outside sources of infection, removing excessive fluid to minimize the risk of seroma and hematoma occurrence, decreasing scar formation and finally preventing unnecessary pain and discomfort.

## Materials and Methods

**Figure 1:**
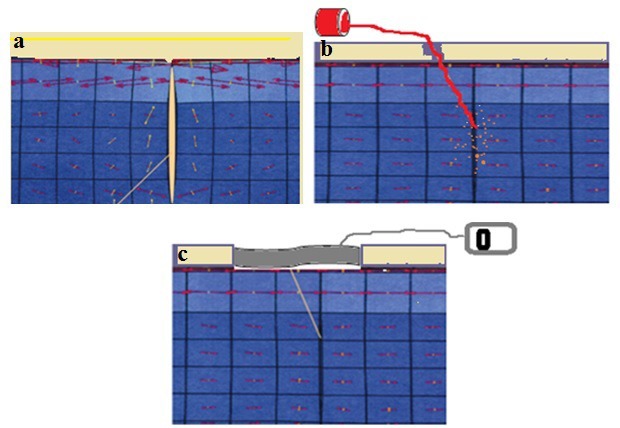
Wound management models; standard dressing model (a), aspiration drainage model (b), negative-pressure wound treatment model (c).

**Figure 2:**
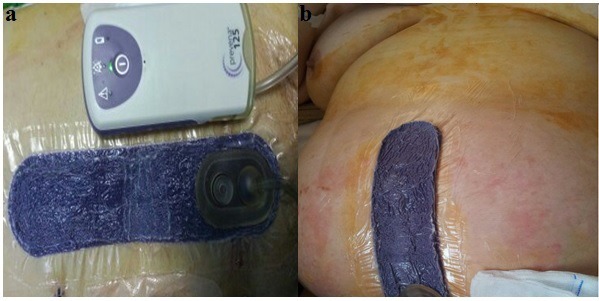
Application of negative-pressure wound therapy (a,b).

In this prospective, randomized, controlled study, we tried to evaluate all patients that were subjected to surgical procedures, with risk factors for wound site problems according to the National Nasocomial Infection Surveillance Committee (NNIS) and nosocomial infections. These factors are represented by contaminated wounds, procedures longer than 2.5 hours, obesity, diabetes or chronic obstructive lung disease, high ASA scores, smoking, malnutrition and immunosuppression. Sixty patients were included in our study, and all of them underwent general surgery procedures. Gynecological or pregnant patients, relaparotomy candidates and patients scheduled for palliative operations were excluded from the study. Three groups were randomized using a computer-generated system. Standart dressings were received by the patients of the control group. In this group of patients, dressings were changed, if necessary, 48 hours after the surgery. Group two received aspiration drainage, where drains were applied to the subcutaneous space (50cc negative-pressure system, Bicakcilar, Istanbul, Turkey) but standard dressings were utilized as well (the models are depicted in Figure 1). All drains were removed when the amount of daily discharge was lower than 5cc. Negative-pressure incision management system (Figure 2) was used for the third group of patients. It was administered right after the procedure on the operating table (KCI, Prevena incision management system, USA). We used this system according to the instruction manual, and the incisions were inspected daily through transparent dressings. The study was terminated on the seventh day, if any problem existed. Demographic data, indications for surgery, BMI, diabetes, presence of COPD, smoking, ASA scores, malnutrition, malignancy and the immune suppression degree were evaluated preoperatively. Postoperatively, wound dehiscence, time of hospital stay, laboratory data [C- Reactive protein (CRP), White Blood Cell (WBC)] and local signs of surgical site inflammation (endurance, infective discharge, pus and warm skin) were noted. Informed consent was obtained from patients who participated in this study, and the authors declare that the research was conducted according to the principles of the World Medical Association Declaration of Helsinki “Ethical Principles for Medical Research Involving Human Subjects”.

### Statistical Analysis

The Statistical Package for the Social Sciences (SPSS 20.0, Chicago, IL, USA) was utilized for data analysis. The mean values, standard deviation, frequency and rates were calculated through the Chi-squared test, Independent Samples T-test, General Linear Model Repeated Measure and One-way ANOVA (p<0.05).

## Results

Sixty-six patients were initially included in our study. However, two patients were excluded due to inoperability, three due to previous operations and one because of the reluctance to participate in the research. The remaining 60 patients were randomized into three groups, of 20 patients each. The mean age was 64.3±8.9 (46-85) but had no statistical significance (p=0.492), and there were 31 (51%) male patients (p=1.54) (Table 1a).

**Table 1a:** Gender and age distribution of patients.

Gender	n	Mean age	Standard deviation	p
Female	29	61.59	10.270	*1.54*
Male	31	66.32	6.983	

Preoperative diagnoses can be found in Table 1b, but there was no statistical difference between the three groups (p=0.517). There was also no difference between elective and urgent surgical procedures (p=1.17) (Table 2a).

**Table 1b:** Distribution of patients’ diagnosis.

Diagnosis	Group			Total (%)
	NP (%)	SD (%)	AD (%)	
Stomach cancer	5 (25)	1 (5)	2 (10)	10 (16.6)
Diseases of the colon and rectum	10 (50)	4 (20)	1 (5)	34 (56.6)
Hepato-biliary diseases	3 (15	12 (60)	5 (25)	6 (10)
Mechanical bowel obstruction	2 (10)	12 (60)	3 (15)	10 (16.6)
**Total**	20 (100)	20 (100)	20 (100)	60 (100)

SD: Standard Dressing (Control group), AD: Aspirative Drainage, NP: Negative Pressure

**Table 2a:** Emergency and elective operations.

	Group			Total (%)	p
	NP (%)	AD (%)	SD (%)		
Emergency	4 (20)	6 (20)	5 (35)	15 (25)	*0.317*
Elective	16 (80)	14 (80)	15 (65)	45 (75)	
**Total**	20 (100)	20 (100)	20 (100)	60 (100)	

SD: Standard Dressing (Control group), AD: Aspirative Drainage, NP: Negative Pressure

Preoperative BMI comparisons and ASA scores were equal (p=0.136 and p=0.115) (Table 2b, 3a). There was no statistical difference between the groups related to COPD, smoking, or diabetes (Table 3b, 4a, 4b).

**Table 2b.** Distribution of patients’ preoperative BMI.

	n	Mean	Standard deviation	p
NP	20	33.05	10.26	*0.136*
AD	20	29.15	5.41	
SD	20	29.00	4.25	
**Total**	60	30.40	7.26	

SD: Standard Dressing (Control group), AD: Aspirative Drainage, NP: Negative Pressure

BMI: Body Mass Index

**Table 3a:** Distribution of patients’ ASA scores.

ASA	Group			Total	p
	NP (%)	SD (%)	AD (%)		
2	1 (51.3)	0 (0)	9 (45)	1 (1.6)	*0.115*
3	8 (20.5)	0 (0)	8 (40)	31 (51.6)	
4	11 (20.5)	11 (55)	20 (100)	28 (46.6)	
**Total**	20 (100)	20 (60)	20 (100)	60 (100)	

SD: Standard Dressing (Control group), AD: Aspirative Drainage, NP: Negative Pressure

**Table 3b:** Distribution of COPD presence.

COPD (Chronic obstructive pulmoner disease)	Group			Total (%)	p
	NP (%)	AD (%)	SD (%)		
Positive	4 (20)	4 (20)	7 (35)	15 (25)	*0.317*
Negative	16 (80)	16 (80)	13 (65)	45 (75)	
**Total**	20 (100)	20 (100)	20 (100)	60 (100)	

**Table 4a:** Distribution of smoking.

Smoking	Group			Total (%)	p
	NP (%)	AD (%)	SD (%)		
Positive	7 (35)	4 (20)	5 (25)	16 (26.6)	*0.764*
Negative	13 (65)	16 (80)	15 (75)	45 (73.4)	
**Total**	20 (100)	20 (100)	20 (100)	60 (100)	

SD: Standard Dressing (Control group), AD: Aspirative Drainage, NP: Negative Pressure

**Table 4b:** Distribution of diabetes mellitus.

DM	Group			Total (%)	p
	NP (%)	AD (%)	SD (%)		
Positive	4 (20)	6 (30)	5 (25)	15 (25)	*0.103*
Negative	16 (80)	14 (70)	15 (75)	45 (75)	
**Total**	20 (100)	20 (100)	20 (100)	60 (100)	

SD: Standard Dressing (Control group), AD: Aspirative Drainage, NP: Negative Pressure

DM: Diabetes Mellitus

Nonetheless, there was a statistical difference regarding hospital stay (p=0.001) (Table 5a).

**Table 5a:** Distribution of hospitalization length.

	n	Mean	Standard deviation	p
NP	20	8.95	2.856	*0.001*
AD	20	14.30	6.689	
SD	20	16.45	7.466	
**Total**	60	13.23	6.715	

SD: Standard Dressing (Control group), AD: Aspirative Drainage, NP: Negative Pressure

Patients in group 3 showed less wound dehiscence compared to the other groups (p=0.017) (Table 5b).

**Table 5b:** Distribution of wound dehiscence.

Wound dehiscence	Group			Total (%)	p
	NP (%)	AD (%)	SD (%)		
Positive	1 (5)	6 (30)	7 (35)	14 (23.3)	*0.017*
Negative	19 (95)	14 (70)	13 (65)	46 (76.6)	
**Total**	20 (100)	20 (100)	20 (100)	60 (100)	

SD: Standard Dressing (Control group), AD: Aspirative Drainage, NP: Negative Pressure

Postoperative WBC and CRP results showed no difference between the three groups (Table 6a, b).

**Table 6a:** Distribution of patient’s leukocyte values.

	n	3^th^ day mean (1000)	5^th^ day mean (1000)	7^th^ day mean (1000)	p
NP	20	10.5±2.76	9.50±2.76	9.80±2.67	*0.128*
AD	20	11.85±2.65	10.26±2.56	9.85±2.37	
SD	20	11.05±2.78	9.05±2.87	8.05±2.15	
**Total**	60	10.87±2.63	9.08±2.67	9.13±2.87	

SD: Standard Dressing (Control group), AD: Aspirative Drainage, NP: Negative Pressure

**Table 6b:** Distribution of patients’ CRP values.

	n	Mean (mg/lt)	Standard deviation	p
NP	20	9.50	2.763	*0.127*
AD	20	10.85	2.159	
SD	20	11.05	2.781	
**Total**	60	10.47	2.633	

SD: Standard Dressing (Control group), AD: Aspirative Drainage, NP: Negative Pressure

CRP: C- Reactive Protein

The negative-pressure incision management system was applied right after the procedure when the patient was still on the operating table and continued for seven days, and effective healing was detected. However, the study was terminated in the case of a single patient due to wound dehiscence on the 5th day. Standart wet dressings were received instead and wound healed by secondary intention on the 12th-day after the intervention. During negative-pressure treatment, foam contamination was avoided, and the wound edges were evaluated continuously in order to detect any erythema. If no abnormality was detected, the procedure continued.

There was no mortality related to the negative-pressure system in our study. One patient had anastomotic dehiscence, and we believe that the cause of death was due to sepsis that occurred as a consequence of the fistula. We do not think the negative pressure system was involved in any way.

However, there are some limitations to our study. Since it is the first one that evaluates the negative pressure incision management system, previous power calculations were absent. Additionally, cost-effectiveness studies were not done, hence the inability to make these types of predictions in our country. Coughing, increased abdominal distention, benign prostatic hyperplasia and other causes of tension were observed in our study, but the inability to measure abdominal pressure remains a significant obstacle for adequate and objective evaluation.

## Discussion

Surgical site infections (SSI) and wound dehiscence remain a major obstacle for successful surgical procedures, causing slow healing times, prolonged hospital stay, and additional operations. Local infection of the operative field is usually prevented by using an aseptic technique and using appropriate antibiotics. SSIs are results of bacterial contamination of the surgical area and have proven to be very expensive, in addition to mortality and morbidity and decreased patient satisfaction and quality of life the corresponding side effects of SSI. Because of the frequency and severity of SSI, high mortality and morbidity rates can be detected. 30% of postoperative mortality is related to SSI, but the type of surgical procedures directly affects SSI rates. In Europe, approximately 30 million operations are performed yearly, and SSI rates in these procedures are nearly 2.6%. In the case of SSI, the mean hospital stay prolongs to 6.5 days, hospital costs increase threefold, and mortality risk increases 2-11 times. Another report explains that the SSI rates in the USA are as high as 500.000 per year. They found that SSI is responsible for 77% of these deaths and hospital costs can raise to 10 billion dollars. Even though there are guidelines to prevent SSIs, it is essential to develop new tools to improve wound healing [3,4].

Co-morbidities (obesity, diabetes, COPD, malnutrition and malignancy) and additionally contaminating impacts of surgical procedures is usually related to wound dehiscence. Wound closure and maintenance techniques play significant roles in wound healing. Therefore, a negative-pressure incision management system is worth to be evaluated in details. Current studies related to this system were mostly performed by orthopedic surgeons, cardiovascular, gynecologists and thoracic surgeons [5–7]. The present study is one of the few studies performed by general surgeons to evaluate the effects of a negative-pressure incision management system. Therefore, there are limited reports in the literature for comparison

Sutures, staples, tissue adhesives and combinations of these are frequently administered for the closure of surgical incisions. Sutures and staples are responsible for tension due to focusing the radiating power to minimal points on the incision site. These points can cause ischemia, infection and tissue necrosis, which can result in wound dehiscence. We believe that negative-pressure incision management systems could be useful in supporting wound healing through suction. Computerized models, in vitro models, animal studies and clinical studies paved the road in describing the effectivity of these systems through holding incisions margins together, decreasing the radiation power, removing excessive fluids, responsible for the accumulation and enriched environment for microorganisms and preventing incisions due to external factors [8–13].

In a simulated computer bench model, the power to separate incision edges 10 mm from each other is compared with a negative-pressure incision management system. Regular incisions were closed with staples. The power required to separate edges 10 mm from each other is 59 % higher when using a negative pressure system (64 Newton vs. 40 Newton). Additionally, the power for full separation in a negative-pressure system is 39% higher (92 Newton vs. 66 Newton). Computer and bench models also have proven that surgical incision management with a negative pressure system immediately decreases lateral tissue tension and increases incision apposition. Better apposition is already known to improve wound healing [14–17].

Stannard et al. prospectively sought to answer whether negative-pressure wound therapy (NPWT) would improve surgical incision healing and hematoma resorption after high-energy trauma where the differences between pressure dressing and vacuum-assisted closure (VAC) were investigated in an attempt to seek for the effects of NPWT in draining hematoma following trauma. Standard postoperative dressing and VAC were used for suturing calcaneal, pilon, and high-energy tibial plateau fractures. Forty-four patients were enrolled in the hematoma study. Drainage was performed in group A and group B for a mean of 3.1 and 1.6 days, respectively (p=0.03). Group A and Group B were infected at rates of 16% and 8%, respectively. The fracture study additionally randomized 44 patients. Group A and Group B also significantly differed with respect to drainage days (4.8 vs. 1.8 days, respectively; p=0.02). Infection and breakdown rates in the present study were not significantly different between the study groups. So far, NPWT has been reportedly used in many complex traumatic lesions. It is thought to boost angiogenesis, increase blood flow, and reduce interstitial fluid. This study still randomizes patients, and so far, it has shown better outcomes with respect to reduced drainage and improved wound healing in both hematomas and severe fractures [18].

In another study, Stannard et al. explored the role of NPWT in the prevention of wound dehiscence and infection following high-risk trauma to the lower extremity. This randomized, multicenter, clinical trial that prospectively investigated blunt trauma patients with either of the three high-risk fracture types (tibial plateau, pilon, calcaneus) with the need for surgical stabilization. Incisional NPWT was applied to patients with closed surgical incisions (Group B, n=141) while standard postoperative dressings were received by the control group (Group A, n=122). The two groups did not significantly differ with respect to the distribution of calcaneus (39%), pilon (17%), or tibial plateau (44%) fractures. There were 23 infections in Group A and 14 in Group B, the difference being statistically significant (P = 0.049). Control subjects had a 1.9 times higher risk of infection than the NPWT patients (95% confidence interval, 1.03-3.55). Therefore, when applied to surgical incisions after the closure of high-risk fractures, NPWT has a favorable effect on wound dehiscence and infections. Furthermore, acute infections were also significantly reduced by NPWT. These data suggest that NPWT is a good option for high-risk wounds following severe skeletal injury [19].

Gomoll et al. retrospectively analyzed 35 patients following the evaluation of negative-pressure on cleanly closed incisions after hip arthroplasty, fixation of proximal femur fracture, and traumatic foot surgery. The team did not detect any infections at three months after using the negative-pressure system for three days [20].

Atkins et al. studied sternal wound infections (SWI) among 57 patients who underwent cardiac surgery and who were deemed at high risk for SWI according to the available risk stratification algorithms. A perioperative risk assessment was followed by surgery and subsequent NPWT application to the clean, closed sternotomy lasting for four days. The researchers recorded all postoperative complications, i.e., SWI, need for readmission, and other complications as well. Coronary artery bypass (CAB) after single internal mammary artery (IMA) grafting was performed in 50.9% of the patients, followed in frequency by combined CAB/valve, non-CAB surgery, and CAB with bilateral IMA. The predicted SWI risk was 6.1 +/- 4%. NPWT could be completed in all the patients problem-free. Death occurred at a rate of 1.8% both at thirty days and during the hospital stay, but it was no related to SWI. SWI did not necessitate any treatment. Although three postoperative SWIs were expected in such a risky cohort, they were possibly eliminated by NPWT, which is an easy-to-use, well-tolerable technique that may potentially improve the wound healing process. In the case of a high SWI risk, NPWT should be strongly considered to be used as a “well wound” therapy [7].

In our study, surgical site infection was detected five times less in the negative-pressure group, compared to the standard dressing group. It is also 3.5-fold less compared to the aspiration drainage group. Similar results were found when compared to wound dehiscence. Wound dehiscence was eight times less in the negative-pressure group and six times less in the drainage group. Hospitalization time was eight days less in the negative-pressure group, compared to the standard dressing one. As we previously mentioned above, insufficient data in the literature remains a major obstacle for an accurate evaluation, due to be initial study of our experiment.

Additionally, we also tried to document contaminated and dirty wounds, whereas other study groups commented on clean wounds. Therefore, our study is the first reporting data regarding this topic. Besides high risks of mortality and morbidity, SSIs are a national problem, occurring in an estimated 2.8 percent of all procedures in our country. For certain types of operations, SSI rates of up to 11 percent have been reported. These infections, which result in complications ranging from redness around the incision to serious cases of sepsis, are also costly, adding an estimated seven days to a patient’s hospital stay. Like many other complications, SSIs can be prevented by following seemingly simple steps that are backed by scientific evidence. However, the complex systems of health care delivery, as well as workplace cultures, often make it a practical challenge to follow some of these steps.

## Conclusion

A negative-pressure incision management system is an easy and fast applicable technique. Negative-pressure delivery through foam decreases lateral tension and edema. This method keeps the incision edges together and reduces the possibility of wound dehiscence. It is an easy and practical method with the advantage of bedside applicability in a sterile environment. It also helps the absorption of exudative fluids and increases blood flow. It facilitates dry surroundings and easily mobilization of the patients. Also, a higher level of job satisfaction is reported among health professionals compared to standard dressings. Surgical site infections and wound dehiscence are tolerable in contrast to other methods. We can conclude that a negative-pressure incision management system is a highly effective and reliable method, especially in risk groups for abdominal closure, increasing patient’s comfort as well. Future studies with more significant numbers are required to pave the road for successful application of negative-pressure systems.

## Ethics Statement

Informed consent was obtained from patients who participated in this study.

## Conflict of Interest

The authors declare there is no conflict of interest.
